# Memory and the hippocampal formation following pediatric traumatic brain injury

**DOI:** 10.1002/brb3.832

**Published:** 2017-11-02

**Authors:** Dana DeMaster, Chad Johnson, Jenifer Juranek, Linda Ewing‐Cobbs

**Affiliations:** ^1^ Department of Pediatrics Children's Leaning Institute University of Texas McGovern Medical School Houston TX USA

**Keywords:** hippocampal development, memory, traumatic brain injury

## Abstract

**Introduction:**

Previous research indicates disruption of learning and memory in children who have experienced traumatic brain injury (TBI).

**Objective:**

This research evaluates the impact of pediatric TBI on volumetric differences along the long axis of the hippocampus, a region of the brain that is critical for explicit memory.

**Methods:**

Structural brain data and behavioral measures were collected 6 weeks following TBI or extracranial injury (EI), in children aged 8–15 years and from a group of age matched typically developing controls (TDC). Total hippocampal volume and hippocampal subregion volumes corresponding to hippocampal head, body, and tail were compared across groups and were examined in relation to verbal and visual memory.

**Results:**

Group differences were evident such that hippocampal body volume was found to be smaller for TBI and EI groups compared to the TDC group. Analysis restricted to the TBI group indicated that hippocampal head volume was associated with severity of injury. The relation between severity of injury and hippocampal head volume is particularly important considering results from our investigation of hippocampal volume‐to‐memory performance relations indicating positive correlations between hippocampal head volume and performance on memory measures for both the TBI group and the TDC group. Significant negative correlations between hippocampal body volume and memory were evident for the TBI group but not EI or TDC groups. Correlations between memory performance and hippocampal tail volume were not significant for the TBI or TDC groups, although for the EI group, a positive correlation was found between hippocampal tail volume and memory.

**Conclusion:**

Together these results underscore an important relation between hippocampal structure and memory function during the subacute stage of recovery from pediatric TBI.

## 1 INTRODUCTION

Memory is one of several important cognitive aptitudes affected by pediatric traumatic brain injury (TBI). TBI sustained during the childhood or adolescent years is particularly concerning because of the potential for disruption in the typical course of brain development and the cascading effects on other cognitive aptitudes. Following moderate to severe TBI, the ability to form and retrieve lasting memories is disrupted and this disruption persists for years following the injury (Babikian et al., 2011). Furthermore, previous research indicates that TBI sustained during childhood and adolescence has strong negative implications for academic success (Arroyos‐Jurado, Paulsen, Ehly, & Max, 2006; Ewing‐Cobbs, Fletcher, Levin, Iovino, & Miner, 1998; Ewing‐Cobbs et al., 2006). Disruption in memory resulting from pediatric TBI has been specifically linked to adverse impact on academic outcomes (Arnett et al., 2013; Ewing‐Cobbs et al., 2004; Fulton, Yeates, Taylor, Waltz, & Wade, 2012; Kinsella et al., 1997). Despite the importance of memory for learning and daily functioning, the mechanisms underlying post‐TBI memory impairment remain under investigated. The goal of the present research was to determine the impact of pediatric TBI on verbal and visual memory in relation to volume of the hippocampus, a region of the brain that is critical for explicit memory.

### 1.1 Structural development of the hippocampal formation in relation to memory

Developmental findings related to structural change in the hippocampal formation were initially mixed with some reports indicating stability in hippocampal volume after the early childhood years (Giedd et al., 1996; Yurcelun‐Todd, 2003) and others showing age‐related change in hippocampal volume through adolescence (Gogtay et al., 2006; Østby et al., 2009). However, in more recent years, results from both longitudinal and cross‐sectional MRI research show regionally specific development along the anterior to posterior axis of the hippocampus (Daugherty, Bender, Raz, & Ofen, 2016; DeMaster, Pathman, & Ghetti, 2013; Gogtay et al., 2006; Schlichting, Guarino, Schapiro, Turk‐Browne, & Preston, 2017). Broadly, results from these investigations consistently report that compared to adults, children show larger volume of anterior segments and smaller volume in posterior segments of the hippocampus formation (Daugherty et al., 2016; DeMaster et al., 2013; Gogtay et al., 2006; Schlichting et al., 2017; also see Insausti, Cebada‐Sánchez, & Marcos, 2010).

Several investigations of regional change in hippocampal volume have focused on relations between age and volume of hippocampal *subregions* along the anterior to posterior axis of the hippocampus roughly corresponding to head, body, and tail regions (DeMaster et al., 2013; Schlichting et al., 2017). In one such cross‐sectional investigation including participants between the ages of 6–30‐year, Schlichting et al. (2017) found an increase in volume of the hippocampal head extending into adolescence which was followed by a decrease in volume between adolescence and middle adulthood. Furthermore, Schlichting et al. (2017) found the converse developmental pattern of volume change in the hippocampal body with a decrease in volume extending beyond adolescence followed by volume increase in the adult years. Although MRI is not sensitive enough to identify the exact maturational processes at a microstructural level, these opposing U shape curves in head and body hippocampal volumes suggests that beyond childhood there are active developmental processes related to synaptic pruning in hippocampal head regions whereas processes related to proliferation might be more localized to the hippocampal body and tail (see DeMaster et al., 2013; Gogtay et al., 2006).

In addition to these *age*‐related differences, recent findings also indicate that hippocampal volume is modulated by common variation in childhood experience such as frequency of aerobic activity, overall fitness, early maternal support, and household income (Chaddock et al., 2010; Herting & Nagel, 2012; Luby, Belden, Harms, Tillman, & Barch, 2016; Rao et al., 2010; Staff et al., 2012; but see Hassevoort, Khan, Hillman, & Cohen, 2016, for a review). Considering findings showing that the hippocampus continues to develop into adolescence with findings indicating that hippocampal development might be susceptible to common variation in environment, it is important to evaluate variation in hippocampal development in relation to neuropsychological outcomes related to memory.

Hippocampal development during childhood is associated with important age‐related increases in episodic memory which refers to the ability to form and later retrieve contextually rich memory for a previously experienced event (DeMaster & Ghetti, 2013; DeMaster, Pathman, Lee, & Ghetti, 2014; DeMaster et al., 2013; Ghetti, DeMaster, Yonelinas, & Bunge, 2010; Lee, Ekstrom, & Ghetti, 2014). However, relations between hippocampal volume and episodic memory in childhood remains a topic of ongoing debate in the literature with some reports indicating smaller hippocampal volumes are cognitively adaptive during childhood, whereas others report the reverse (for a review, see Van Petten, 2004; Østby, Tamnes, Fiell, & Walhovd, 2012). Indeed, in developing populations, positive relations between volume of the entire hippocampal structure and memory performance are evident (Østby et al., 2012). When volume of the hippocampus is calculated for each subregion, results indicate that in adults, the hippocampal head is negatively correlated with memory performance (DeMaster et al., 2013; Poppenk & Moscovitch, 2011). A negative association was also found by Schlichting et al. (2017) between performance on an inferential learning task and hippocampal head volume in children, adolescents and adults. In contrast to the negative relations reported between hippocampal head volume and memory performance, in a younger group of children a positive correlations between bilateral hippocampal head volumes and episodic memory performance for a group of 6‐year‐olds, although no correlations were evident for a comparison group of 4‐year‐olds (Riggins, Blankenship, Mulligan, Rice, & Redcay, 2015). In more posterior regions of the hippocampus corresponding to the hippocampal body and/or tail, adults show a positive correlation between memory performance and volume of the body (DeMaster et al., 2013) and the body and tail (Poppenk & Moscovitch, 2011); whereas, DeMaster et al. (2013) found a positive correlation between hippocampal tail volume and memory performance in children aged 8–11 years.

Taken together results from volumetric investigations of the hippocampus suggest a link between hippocampal development in childhood and age‐related increase in memory in healthy populations. However, it remains unclear how TBI sustained during childhood disrupts the normal course of development of the hippocampus, and whether disruption of hippocampal development results in impaired memory aptitude.

### 1.2 Memory after TBI: relation to hippocampal volume

The behavioral aspects of pediatric TBI‐induced memory deficits have been an area of active research for many decades (Campbell, Kuehn, Richards, Ventureyra, & Hutchison, 2004; Farmer et al., 1999, 2002; Levin, Eisenberg, Wigg, & Kobayashi, 1982; Levin et al., 1988). Indeed, there is evidence that a wide range of memory functions are impacted by moderate to severe pediatric TBI, with child injury groups performing worse than age‐matched comparison groups on standardized tests of general memory, visual memory, and learning (Farmer et al., 1999; Thaler, Barney, Reynolds, Mayfield, & Allen, 2011). Specifically, results from Thaler et al. (2011) indicate that children with a history of TBI demonstrated deficits on tests of verbal recall, object recall, spatial recall, and memory for faces. Furthermore, deficits in memory functioning following pediatric TBI are long lasting (Horneman & Emanuelson, 2009) and increase with severity of injury (Levin et al., 1988).

Taken together, these behavioral findings suggest that moderate to severe TBI often results in pervasive and chronic memory deficits. Historically, decreased memory function in TBI populations has been reported to reflect deficits in monitoring and control processes that result from damage sustained to PFC regions (Di Stefano et al., 2000). Considering that maturation of PFC continues into early adulthood and that PFC development mediates age‐related increase in memory‐related monitoring and control processes (Ofen, 2012; Ofen, Chai, Schuil, Whitfield‐Gabrieli, & Gabrieli, 2012), TBI‐related insult to PFC during childhood likely results in long‐term deficits in memory functioning (Phillipou, Douglas, Krieser, Ayton, & Abel, 2013). However, in addition to deficits related to damage to PFC, there is growing evidence that the hippocampal formation is also adversely affected by TBI (Ariza et al., 2006; Tasker et al., 2005; Wilde et al., 2007). Investigations that evaluated young adults in the years following injury consistently report reductions in hippocampal volume following TBI, particularly for those with moderate to severe injury (Palacios et al., 2013; Rushby et al., 2016; Zagorchev et al., 2016; although see Ariza et al., 2006). Although more recently, results from a longitudinal study indicate decreased hippocampal volume following TBI that was mild in severity rather than moderate to severe (Zagorchev et al., 2016).

Traumatic brain injury‐related hippocampal volume reductions are widely considered to result from atrophy occurring throughout the structure (Royo et al., 2006). However, there is some evidence suggesting that, in young adults, TBI‐related injury to the hippocampus is selective, with disproportionate insult to anterior compared with posterior hippocampal subregions when measured 6–8 months postinjury (Ariza et al., 2006). An additional investigation to examine the impact of TBI in adulthood combined volumetric measures with measures of neuronal integrity estimated from diffusion tensor imaging (Avants et al., 2008). Using this multimodal approach, results from Avants et al. (2008) indicate a convergence in reduction in volume with decreased neuronal integrity localized in the anterior hippocampus. Taken together these findings indicate that rather than diffuse injury, which would impact the entire hippocampal formation, in the months following injury, TBI may selectively affect anterior regions of the hippocampus corresponding to the hippocampal head.

The implication of TBI for hippocampal *development* in child populations remains under‐investigated, although there are some initial reports suggesting that TBI sustained in childhood might disrupt the course of hippocampal maturation (Serra‐Grabulosa et al., 2005; Tasker et al., 2005; Wilde et al., 2007). Specifically, in youth from 9 to 16‐years who sustained a TBI at least 1 year prior to evaluation, Wilde et al. (2007) reported smaller hippocampal volume bilaterally compared to age‐matched healthy controls. Of interest, Wilde et al. (2007) also found that the hippocampus volume was disproportionately decreased compared to other subcortical structures such as the amygdala, suggesting that the hippocampus is particularly vulnerable to pediatric TBI.

Given that there is substantial evidence that the hippocampus is critical for the vast majority of memory‐related behaviors and is highly vulnerable to injury resulting from TBI, it is not surprising that a handful of investigations have shown association between reduced memory performance and hippocampal atrophy following a TBI (Ariza et al., 2006; Bigler, Johnson, Anderson, Blatter, & Al, 1996; Dennis et al., 2016; Tate & Bigler, 2000). However, other reports have demonstrated an association between injury‐related cortical thinning and memory performance, but not for the hippocampal formation in children (Di Stefano et al., 2000) or adults (Palacios et al., 2013). Consistent with Ariza et al. (2006), a possible explanation for divergent results might be that some hippocampal regions are more vulnerable to injury than others. Indeed, recent support for selective atrophy of the hippocampal formation following TBI was contributed by Dennis et al. (2016) using tensor‐based morphometry methodology, which is able to capture group differences in brain regional volume *and* shape. Dennis et al. (2016) investigated TBI‐induced structural changes throughout the brain in a sample of older children with a mean age of 14‐years at enrollment. Of interest, although hippocampal volumes were similar to non‐injured peers, results indicate that, for children with a history of TBI, at 1–2 years postinjury, higher cognitive performance was related to smaller volume in the anterior pole of the hippocampus. However, cognitive performance in this study was operationalized as a linear composite of processing speed, working memory, verbal learning, and cognitive switching skills, rather than focusing specifically on memory performance.

The influence of age at the time of brain injury on specific structures and abilities is not well understood. However, it is likely that brain regions and cognitive abilities that are developing rapidly at the time of injury may be particularly vulnerable to disruption (Ewing‐Cobbs, Fletcher, & Levin, 1986; Ewing‐Cobbs et al., 2016). It is therefore likely that the hippocampus, which has been shown to continue to develop into adolescence (DeMaster et al., 2013) and even adulthood (Daugherty et al., 2016), is highly vulnerable to TBI sustained in childhood because injury may disrupt maturation of the hippocampal formation and restrict connections between the hippocampus and other brain structures. However, to the best of our knowledge, the relation between volumetric variation along the long axis of the hippocampus and memory in children with TBI has not been investigated.

Here, we collected high‐resolution structural MRI images and measures of memory from children and adolescents with subacute TBI, extracranial injury which did not result in TBI (extracranial injury [EI]), and a typically developing comparison (TDC) group. We then compared relations between hippocampal volume and performance on tests of memory to determine if group differences were evident in hippocampal‐memory associations. Our investigation was guided by three hypotheses. First, it was hypothesized that pediatric TBI would result in lower performance on memory tests compared to age‐matched healthy controls and an EI group. Second, based on research in adult TBI populations by Ariza et al. (2006), it was expected that TBI‐related atrophy would be evidenced by smaller regional hippocampal volumes, particularly in anterior regions of the hippocampus. Finally, we hypothesized that group differences in relations between hippocampal volume and performance on memory tests will be apparent. Specifically, we expect that the TBI group would show a positive relation between hippocampal volume, particularly in head regions, and memory performance.

## 2 MATERIALS AND METHODS

### 2.1 Participants

A total sample of 129 children, aged 8–15 years, participated in this research including youth with TBI (*n* = 62), EI (*n* = 29), and TDC (*n* = 38). The injury groups (i.e., TBI and EI participants) were recruited from the Level 1 Pediatric Trauma Center at Children's Memorial Hermann Hospital/University of Texas Health Science Center at Houston. The TDC participants were recruited from the same community as TBI and EI participants by advertising with flyers in locations frequented by parents and attending local civic events. The enrollment procedure included steps to match TBI, EI, and TDC groups in age and sex.

Given that previous research indicating that hippocampal volume is modulated by highly prevalent variations in childhood experience (Hassevoort et al., 2016), it is likely that the inactivity and stress that result from sustaining a serious injury might account for variation in postinjury hippocampal volume. It is also possible that preinjury characteristics, such as risk‐taking behavior, could also influence outcomes. To account for these preinjury characteristics and postinjury factors, children with history of EI were included as a comparison injury group. An additional benefit of including an EI patient group is the opportunity to differentiate effects specifically related to injury to the brain from the consequences of injury in general.

All injured participants met the following inclusion criteria: (i) treatment in the emergency department or hospitalization for TBI or EI, (ii) age at injury between 8 and 15 years, (iii) participant proficient in English and parent proficient in English or Spanish, (iv) residing within a 125 mile catchment radius, (v) no preinjury history of major neuropsychiatric disorder such as intellectual deficiency or low functioning autism spectrum disorder that would confound assessment of the impact of injury on imaging or behavioral outcomes, (vi) no prior hospitalization for anxiety or depression, (vii) no history of type 1 or type 2 diabetes, and (viii) no prior medically treated TBI. Typically developing children were recruited from community notices and met inclusion criteria 2–7.

Demographic characteristics of the TBI, EI, and TD participants are provided in Table 1. The injury groups experienced acceleration‐deceleration or blunt impact injuries in motor vehicle accidents. The severity of head injury was rated using the Glasgow Coma Scale (GCS) score collected at hospital admittance (Teasdale & Jennett, 1974), where severe TBI was a score of 3–8 and moderate TBI was a score of 9–12. Complicated‐mild TBI was classified as a GCS score of 13–15 combined with acute hemorrhage or parenchymal injury seen in acute neuroimaging (Levin et al., 2008). Severity of Injury for the EI and TBI groups was measured with the Severity of Injury Score (ISS) (Baker, O'Neill, Haddon, & Long, 1974). ISS is a calculated score for six body regions (head/neck, face, chest, abdomen, extremities, and skin) with a range of ≤9 for minor injuries and ≥24 for severe injuries. We calculated a total ISS score and an ISS score excluding injury to the head which verified that the EI group did not have evidence of trauma to the head or concussion symptoms.

**Table 1:** Demographic and injury information by group

Total sample	Group			Statistic	*p*
	Traumatic brain injury (*n* = 62)	Extracranial injury (*n* = 29)	Typically developing (*n* = 38)		
Months of age, *M* (*SD*)	147.52 ± 26.44	144.69 ± 29.04	147.61 ± 27.28	*F*(2, 126) *= *0.122	.89
Sex, % Male	60	69	61	χ^2^(2, *N = *129) *= *0.77	.68
Maternal education (*n*)					
High school or less	31	19	10	χ^2^(2, *N = *129) *= *10.74	.005
Post high school	31	10	28		
Race (*n*)					
African American	16	3	8	χ^2^(4, *N = *129) *= *5.715	.221
Caucasian	42	26	27		
Other/Multiethnic	4	0	3		
TBI severity (*n*)					
Mild	31				
Complicated mild	11				
Moderate	5				
Severe	15				
Admission Glasgow Coma Score, *M* (*SD*)	12.11 ± 4.16				
Injury Severity Score, *M* (*SD*)	14.33 ± 10.25	10.52 ± 6.24			

### 2.2 Procedure

Participants were recruited during or shortly following the initial hospital visit. The research was conducted in accordance with the Code of Ethics of the World Medical Association, the granting agency, and the University Institutional Review Board. Informed written consent was obtained from the child's guardian according to Institutional Review Board guidelines. Written assent was obtained from all participants prior to data collection.

As part of the follow‐up protocol, 6 weeks after injury, memory measures were administered by a trained research assistant and participants received an MRI scan. For each participant, behavioral testing was conducted on the day of MRI data acquisition prior to scanning.

### 2.3 Tasks

The primary measure of memory consisted of three subtests of the Test of Memory and Learning 2 (TOMAL2), which is an age‐normed memory test designed for children between the ages of 5–19 years (Reynolds & Voress, 2007). Participants completed two TOMAL2 subtests that required immediate retrieval of sequences: (i) Visual Selective Reminding (VSR) and (ii) Word Selective Reminding (WSR Immediate condition). In addition, the Word Selective Reminding Delay (WSR Delay condition) was administered, which involved retrieval of words sequences from the WSR Immediate condition following a 30 min delay. Scaled scores corrected for age were used in all analyses.

During the VSR task, participants were asked to learn a spatial dot pattern over the course of several trials. Participants were given reminders on missed sequences until the pattern was produced correctly, or after five unsuccessful attempts had elapsed. During the WSR Immediate condition, participants were presented with a series of words and asked to repeat the list of words to the examiner. If a participant failed to report a word from the list, the experimenter would provide a reminder for the word and the participant was then asked to begin the list again. This was repeated for six trials or until the list was recalled correctly. Following a delay of 30 min, participants were administered the WSR Delayed condition during which they retrieved and reported the list of words learned during the WSR Immediate condition.

### 2.4 MRI data

Structural brain data were acquired on a research‐dedicated Philips 3T MR scanner with a 32 channel head coil at the University of Texas McGovern Medical School. High resolution T1‐weighted anatomical scans were acquired (TR = 8.1, TE = 3.7, flip angle = 6°, matrix = 256 × 256, slice thickness = 1 mm, and voxel size = 1 × 1 × 1) with a scan duration of 4:47 min. The scanning facility replaced the scanner with a Philips 3T Ingenia toward end of data collection and as a result 13 of our participants were collected after the upgrade. Fidelity analysis was performed to match T1‐weighted scanning protocols but some variations might remain. To account for differences in scanner, we included scanner change as a covariate in all analysis including MRI data.

**Figure 1:**
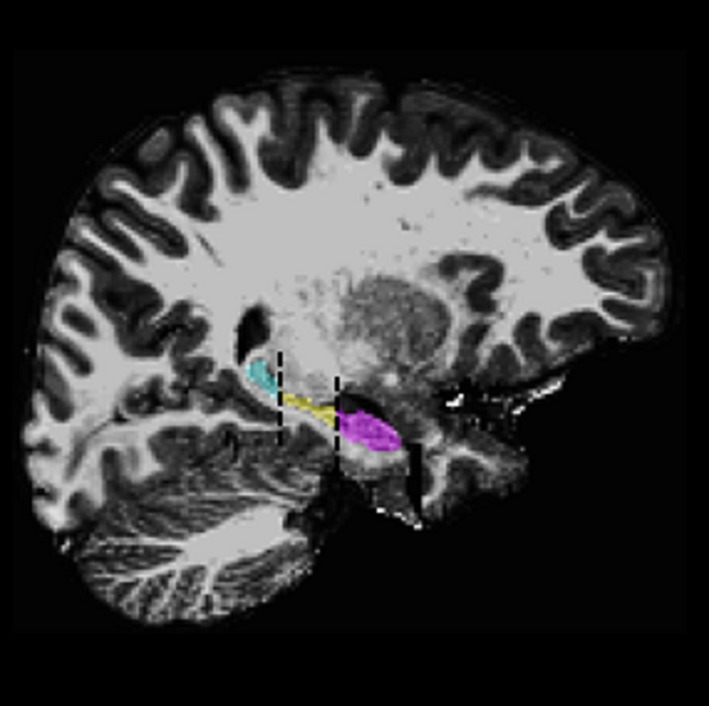
Showing right hippocampal segmentation with FreeSurfer postmanual edits in a traumatic brain injury participant. Hippocampal head in purple; hippocampal body in yellow; hippocampal tail in light blue

Cortical and subcortical volumes were first segmented with FreeSurfer version 5.3.0, an automated segmentation software program (http://surfer.nmr.mgh.harvard.edu). Following pre and postprocessing in FreeSurfer, manual inspection of automated segmentation of the hippocampus was conducted and if required, corrections of hippocampal boundaries were made. To further segment the hippocampal formation along the anterior to posterior axis, the hippocampi were manually parcellated into head, body, and tail regions using the Freesurfer tkmedit tool for visualization. Segmentation of hippocampal regions was done in the coronal plane by an expert in hippocampal anatomy (DD), who followed a previously developed hippocampal segmentation protocol used by DeMaster et al. (2014) and also used by Riggins et al. (2015) based on hippocampal head, body, and tail boundaries as defined in Duvernoy, Cattin, and Risold (2013). Briefly, using this protocol the hippocampal head is segmented from the hippocampal body at the point where hippocampal digitations begin to smooth on the dorsal edge and the hippocampus takes on a tear‐like shape. Moving to posterior hippocampus, the body of the hippocampus is segmented from the tail of the hippocampus at the point where the fornix is visible indicating that the fornix is separating from hippocampus proper. An example of anterior to posterior segmentation is provided in Figure 1.

To verify accuracy of hippocampal parcellation into head, body and tail regions, intra‐rater reliability was conducted on over 50% of cases. Collapsing across left and right, slice selection for hippocampal head and tail was equal or within two slices for 99% of cases. Intraclass correlations were also calculated (Shrout & Fleiss, 1979) for each region and verify highly reliable implementation of the protocol for hippocampal head and tail slice identification. In left and right hemispheres, intraclass coefficient above .98 were evident for both hippocampal head and tail slice identification.

### 2.5 Statistical approach

#### 2.5.1 Group comparisons

To evaluate comparability of demographic variables across groups, an ANOVA was performed to determine if TBI, EI, and TDC groups differed in age. Chi‐square tests of independence were performed to examine if the distribution of maternal education and ethnicity differed across groups and should be considered as covariates (see Table 1).

To assess the effect of group on TOMAL subtest scaled scores, a 3 (Group: TBI, EI, TDC) × 3 (Task: VSR, WSR Immediate Condition, and WSR Delay Condition) repeated‐measures ANCOVA was employed. To evaluate whether total and regional hippocampal volumes differed across groups, we first examined difference in volume of the hippocampal structure as a whole. We conducted at 3 (Group: TBI, EI, TDC) × 2 (Hemisphere: Left, Right) repeated‐measures ANCOVA controlling for maternal education, age, total brain volume, and scanner change. To assess hippocampal subregions, a 3 (Group: TBI, EI, TDC) × 3 (Subregion: Head, Body, Tail) × 2 (Hemisphere: Left, Right) repeated‐measures ANCOVA was completed controlling for maternal education, age, total brain volume, and scanner change.

#### 2.5.2 Correlational analyses

Correlation analyses were conducted to evaluate the effects of injury on memory performance, hippocampal volume, and hippocampal volume‐to‐memory relations. To examine the impact of injury severity on outcomes, the GCS score indexed severity of TBI and the ISS score was used for EI participants. As these are ordinal scales, Spearman Rank order correlations were used to assess injury severity in relation to memory scores as well as total and regional hippocampal volumes.

Finally, Pearson correlations were used to determine if hippocampal volume was related to memory scores for each group separately. For correlation analyses residual scores accounting for maternal education were calculated for each scaled TOMAL2 task score. For hippocampal volume scores, demographic and age effects were accounted for by calculating residual scores for hippocampus as a whole and hippocampal subregion volumes with the effects of maternal education, age, total brain volume, and scanner change removed. Following correlation analyses, Fisher's *z* tests, two‐tailed, were used to evaluate whether significant differences in the hippocampal‐to‐TOMAL2 correlations existed between groups.

## 3 RESULTS

### 3.1 Participants

Results indicated that groups did not differ in age or ethnicity. However, maternal education differed between groups. Previous reports indicate that early maternal support (Luby et al., 2016) and family income (Staff et al., 2012) are related to hippocampal volume in childhood and young adulthood, respectively, and as a result maternal education was included as a covariate in all subsequent analyses.

### 3.2 Behavioral Findings

ANCOVA evaluated group differences in memory controlling for the effect of maternal education. Mauchly's test indicated the assumption of sphericity was violated *x*2 (2) = 24.327, *p* < .005, and thus degrees of freedom were corrected using Huynh‐Feldt estimates of sphericity (ɛ = .880). Results show a main effect of TOMAL2 Task *F*(1.76, 219.20) = 4.198, *p* = .020 and a TOMAL2 Task × Group interaction *F*(3.50, 219.20) = 5.374, *p* = .001. To follow‐up interaction effects, we conducted a separate ANCOVA for each task.

For the WSR Immediate Condition and WSR Delay Condition, ANCOVA results were similar and showed significant effects of group *F*s(2, 124) = >3.418, *p *≤* *.036. Post hoc comparisons using *t* test with Bonferroni correction indicated lower performance for the TBI group compared to the TDC group (*p*s ≤ .038) on both tasks; however, no difference in performance was evident for the EI group compared to either the TBI or the TDC group. Group effects for the VSR task did not reach traditional thresholds of significance *F*s (2, 124) = 2.603, *p* = .078. These results can be seen in Table 2.

**Table 2:** Group means and standard error of scaled scores for each Test of Memory and Learning–Second Edition (TOMAL2) subtest

Memory subtest	Group								
	Traumatic brain injury *n = *62		Extracranial injury *n = *29		Typically developing *n = *38		Statistic (*p‐*Value)		
	*M*	*SE*	*M*	*SE*	*M*	*SE*	TBI vs. EI	TBI vs. TDC	EI vs. TDC
Visual Selective Reminding	10.06	0.33	8.72	0.49	9.72	0.43	.07	1.00	.41
Word Selective Reminding	8.43	0.34	9.74	0.51	10.08	0.44	.10	.01*	1.00
Word Selective Reminding Delay	9.98	0.23	10.58	0.35	10.95	0.30	.47	.04*	1.00

EI, extracranial injury; TBI, traumatic brain injury; TDC, typically developing controls.

Means adjusted for maternal education with difference significant at the .05 level indicated with *. Bonferroni adjustment for multiple comparisons.

To determine if TBI severity was related to performance on the memory measures, Spearman's rank‐order correlation partialling maternal education revealed a trend for GCS scores to correlate with WSR Immediate condition, *r*(62) = .241, *p* = .059. This positive correlation suggests that individuals with higher GCS scores, indicating a lower level of severity, showed higher performance on the WSR Immediate Condition. Correlations between GCS and VSR and WSR Delayed Condition scores were not significant (*p*s = >.143). Spearman's rank‐order correlation revealed that the Injury Severity Score was not related to TOMAL2 subtests for either the TBI or EI groups (*p*s = >.409).

### 3.3 Difference in hippocampal head, body, and tail volume

Results of ANCOVA indicated no significant main effect of Hemisphere *F*(1, 122) = 0.073, *p* = .788 or Hemisphere by group interaction *F*(2, 122) = 0.962, *p* = .385 suggesting total hippocampal volumes were similar between right and left hemispheres and across group.

ANCOVA results indicated a main effect of Hippocampal Subregion *F*(2, 244) = 7.877, *p* = .001 and a Hippocampal Subregion × Group interaction *F*(4, 244) = 4.386, *p* = .001. However, no main effect of Hemisphere *F*(1, 122) = 0.130, *p* = .719 was evident. Mauchly's test indicated the assumption of sphericity was violated for the Hemisphere × Region analysis *x*2 (2) = 50.992, *p* < .005, and thus degrees of freedom were corrected using Huynh‐Feldt estimates of sphericity (ɛ = .788). Following this correction, Hippocampal Subregion × Hemisphere and, most importantly, Group × Hippocampal Subregion × Hemisphere interactions were not significant (*p*s* = >*.254).

Given that no effects of Hemisphere were evident, we created a mean volume score for each hippocampal subregion by averaging left and right volumes. Results corresponding to left and right hippocampus are available in Table S1. Using these mean scores the Hippocampal Subregion × Group interaction was followed‐up with ANCOVA performed separately for each hippocampal subregion and Post hoc comparisons using *t* test with Bonferroni correction. (see Table 3). Group differences were found and indicated larger hippocampal body volume for the TDC group compared to the TBI and EI groups (*p*s ≤ .015) but no difference between hippocampal body volume was evident for the TBI group compared to the EI group. Group differences were not evident for either hippocampal head or tail volumes *F*s (2, 122) ≤ 1.736, *p* ≥ .180.

**Table 3:** Mean and standard error of average of left and right hippocampal head, body, and tail volume

Subregion	Group								
	Traumatic brain injury *n = *62		Extracranial injury *n = *29		Typically developing *n = *38		Statistic (*p‐*Value)		
	*M*	*SE*	*M*	*SE*	*M*	*SE*	TBI vs. EI	TBI vs. TDC	EI vs. TDC
Head	1,728	33	1,767	49	1,648	43	1.000	.438	.238
Body	1,350	29	1,294	44	1,489	38	.882	.015*	.004*
Tail	622	18	630	26	645	23	1.000	1.000	1.000

EI, extracranial injury; TBI, traumatic brain injury; TDC, typically developing controls.

Values adjusted for maternal education, age, total brain volume, and scanner change with difference significant at the .05 level indicated with *. Bonferroni adjustment for multiple comparisons.

To determine if volumetric differences were correlated with TBI severity, the GCS score was correlated with hippocampal subregions. Spearman's correlations controlling for all covariates indicated a positive correlation between GCS and head volume *r*(62) *= *.33, *p = *.009. This finding indicates that individuals with higher TBI severity (lower GCS score) had smaller hippocampal head volumes. Relations between GCS and hippocampal body and tail volume were not evident (*p*s > .432). Spearman's correlations were also performed for TBI and EI groups using the residual scores for hippocampal subregions and Injury Severity Scores. However, relations between Injury Severity Score and hippocampal head, body, and tail volume were not evident for either group (*p*s* = *>.096).

### 3.4 Differences in relations between hippocampal volume and memory

Pearson correlations using residualized TOMAL and hippocampal values were conducted for each group separately. Coefficients for TOMAL2 subtests and hippocampal volumes by group are provided in Table 4; separate coefficients for left and right hippocampus are in Table S2. For brevity only correlations that reach traditional levels of significance for one or more of the groups will be reported in this section.

**Table 4:** Pearson correlation for performance on Test of Memory and Learning–Second Edition (TOMAL2) subtests and average of left and right hippocampal head, body, and tail volume

Group	Hippocampal		
	Head	Body	Tail
Visual Selective Reminding			
Traumatic brain injury	.14	−.12	−.17
Extracranial injury	−.18	−.16	.18
Typically developing	.03	−.19	.09
Word Selective Reminding			
Traumatic brain injury	.30*	−.43** ^,^ a ^,^ b	−.09
Extracranial injury	−.08	.15	.09
Typically developing	.22	.02	−.14
Delayed Word Selective Reminding			
Traumatic brain injury	.27*	−.26*	−.06
Extracranial injury	.23	−.30	.42* ^,^ b ^,^ c
Typically developing	.33*	−.19	−.27

EI, extracranial injury; TBI, traumatic brain injury; TDC, typically developing controls.

Values adjusted for maternal education, age, total brain volume, and scanner change.

^a^TBI ≠ TDC, ^b^TBI ≠ EI, ^c^EI ≠ TDC.

*p ≤* *.05; **p ≤* *.01. Fisher's *r* to *z* transform, *p* < .05.

For the hippocampal head, a significant positive correlation was evident between performance on WSR Immediate condition and hippocampal head volume *r*(62) *= *.295, *p = *.02 for the TBI group but not the EI or the TDC groups. For both the TBI and TDC groups, positive correlations were also evident between performance on the WSR Delayed Condition and hippocampal head volume (TBI group: *r*(62) *= *.272, *p = *.032, TDC group: *r*(38) *= *.330, *p = *.043). Fisher *Z* tests indicated that correlations were not significantly different between groups.

For the body of the hippocampus, correlations between memory performance and hippocampal body volume were negative and only evident for the TBI group. First, a significant negative correlations was evident between hippocampal body volume and performance on the WSR Immediate task *r*(62) *= *−.428, *p = *.001 with Fisher *Z* tests indicating that the correlation was different from both the EI group (Fisher *Z = *−2.59, *p = *.009, two‐tailed) and the TDC group (Fisher *Z = *−2.24, *p = *.03, two‐tailed). A negative correlation was also evident between hippocampal body volume and performance on the WSR Delayed condition *r*(62) *= *−.263, *p = *.04 but this correlation was not found to differ significantly between groups.

Finally, in the tail of the hippocampus a positive correlation was evident between tail volume and performance on the WSR Delayed condition for the EI group condition *r*(29) = .418, *p = *.024 with fisher *Z* tests indicating that the correlation was different from both the TBI group (Fisher *Z = *2.15, *p = *.03, two‐tailed) and the TDC group (Fisher *Z = *2.77, *p = *.005, two‐tailed).

## 4 DISCUSSION

The goal of this research was to determine the impact of TBI on total and regional hippocampal volumes and to characterize relations between memory and hippocampal volumes in children during the subacute stage of recovery from TBI in relation to children sustaining bodily injury and a healthy comparison group. The hippocampus is vulnerable to the effects of TBI (Ariza et al., 2006) and injury sustained during childhood might alter the developmental trajectory of the hippocampal formation (Wilde et al., 2007). To our knowledge this is the first study to investigate differences in volume of the hippocampal head, body, and tail subregions in children with a history of TBI and their association with memory. Results indicate that group membership was related to both memory scores and hippocampal volumes segmented along the anterior to posterior axis. Children with TBI had lower immediate and delayed word recall, but not immediate visual memory scores, compared to either the EI or healthy groups. Although volume of the head and tail did not vary by group, volume of the body was smaller in children with either brain or bodily injury than in the healthy comparison group. The severity of brain injury, as indexed by the GCS score, was positively correlated with hippocampal head volume such that greater TBI severity was associated with smaller head volume. With regard to the memory scores, lower scores on word list learning and delayed recall were positively associated with head volume and negatively associated with body volume only in participants with TBI. Bodily injury showed a positive correlation between tail volume and delayed word list recall. These findings highlight significant relations between regional hippocampal volume and memory performance during the early stages of recovery in children hospitalized following brain or bodily injury.

The pattern of memory scores after pediatric TBI varies in relation to severity of injury. Previous findings from a meta‐analysis of memory scores revealed minimal verbal or visual deficits following mild injuries. Children sustaining moderate or severe TBI showed divergent patterns, with visual immediate memory disproportionately reduced following moderate TBI and vulnerability of verbal immediate and delayed recall following severe TBI (Babikian & Asarnow, 2009). Our sample, which consisted of the full range of TBI severity, also found particular vulnerability of immediate and delayed word memory relative to visual location memory. It was predicted that the TBI group would not perform as well on TOMAL2 subtests compared to individuals with no history of TBI. Results indicate reduced performance on both the WSR Immediate condition and WSR Delayed condition for the TBI group compared to the TDC group a result that is consistent with Thaler et al. (2011). However, Thaler et al. (2011) found an effect of TBI on all TOMAL2 tasks in a child population, whereas our results indicate no difference between groups in performance on VSR tasks. The discrepancy in TOMAL2 effects may result from several methodological differences between studies. Thaler et al. (2011) reported on TOMAL2 scores from a large retrospective sample of convenience with limited characterization of TBI severity. The apparent greater injury severity and longer injury to test interval may have increased detectability of TBI‐related deficits in performance on memory measures. It should also be noted that performance on Tomal2 subscale tasks did not differ between the TBI and EI groups suggesting the possibility that sustaining an injury to any location on the body might be a disruptive factor in memory development.

Estimating volume of the hippocampal formation as a whole, Wilde et al. (2007) found that, compared to healthy controls, children with a history of TBI showed reduced hippocampal volume 3 years postinjury. In this study, no group differences were evident in volume of the entire hippocampal formation when evaluated 6 weeks postinjury. The longer period postinjury and increased level of severity of injury in the TBI group included in Wilde et al. (2007) likely accounts for divergent results. However, the procedure used to identify hippocampal boundaries might also account for our results indicating more normative total hippocampal volumes in the TBI group. Specifically, compared to our use of Freesurfer to isolate the hippocampus, it is possible that manual tracing of hippocampal boundaries, used by Wilde et al. (2007), was better able to capture injury related atrophy.

Whereas our results indicate no group differences in volume when the hippocampus was evaluated as a whole, injury‐related reduction in volume was evident when the hippocampus was segmented into subregions along the anterior to posterior axis. Results corresponding to hippocampal subregions indicated smaller hippocampal volumes related to injury in the body of the hippocampus suggesting that segmenting the hippocampal formation into subregions facilitates identification of injury‐related reductions in volume. Based on Ariza et al.'s (2006) results indicating atrophy in the hippocampus that was localized to hippocampal head, it was surprising that volume reduction in this study was evident in the body rather than the head of the hippocampus. Since Ariza et al. (2006) included TBI patients with a moderate to severe injury, whereas our sample ranged in severity from mild to severe; it is likely that reduction in hippocampal head volume might be more evident in individuals with severe rather than mild injury. To address this possibility, we examined whether severity of injury was correlated with hippocampal volume. Smaller volume of the hippocampal head, but not more posterior hippocampal regions, was associated with an increase in TBI severity. Consistent with Ariza et al. (2006), these finding suggests that similar to adult TBI populations, severe TBI sustained during childhood may result in reduction in hippocampal head volume. Overall, these results provide converging evidence that the hippocampal head is more vulnerable to damage from TBI than more posterior regions of the hippocampus.

While beyond the scope of this research, it is also valuable to consider how cytoarchitectural subfields of the hippocampus corresponding to dentate gyrus (DG), the cornu ammonis (CA) subfields CA3, and CA1 might influence the present results. Drawing from hippocampal subfield literature, one reason to expect increased vulnerability of the hippocampal head region is that subfield CA1 volume is proportionally greater in the hippocampal head compared to body and tail hippocampal regions (Duvernoy, Cattin, & Risold, 2013). Furthermore, subfield CA1 has been shown to be highly sensitive to cell damage resulting from TBI (Casella et al., 2014; Maxwell et al., 2003) and results indicate that CA1 plays a substantial role in memory for sequential events over long periods of time (Farovik, Dupont, & Eichenbaum, 2010). Memory for the sequence of events is one of the hallmarks of episodic memory. The possibility of increased risk of damage to CA1 from TBI in relation to sequential memory should be further examined in the pediatric TBI population.

MRIs obtained for this study did not have the required resolution to isolate hippocampal subfields; however, our results indicated a relation between larger hippocampal head volume and increased performance on the WSR Delayed tasks for both the TBI and TDC group and for the WSR immediate task for the TBI group. This finding is consistent with the positive correlation between hippocampal head volume and memory previously reported in healthy young children (Riggins et al., 2015). In contrast to positive correlations evident in the hippocampal head, the TBI group showed negative correlations between hippocampal body volume and performance on both WSR Immediate condition and WSR Delayed condition. Fisher's *Z* test indicated that the correlation between hippocampal body volume and performance on WSR Immediate condition found in the TBI group was significantly different from the TDC and EI groups. This finding, in conjunction with historical findings indicating smaller anterior hippocampal volume associated with higher memory performance (see DeMaster et al., 2013; Maguire, Woollett, & Spiers, 2006) suggests that variations along the anterior to posterior axis of the hippocampus involving both the head and body are evident following TBI and likely result in memory impairment.

Results from this investigation suggest that the relation between hippocampal morphology and memory may be altered following a TBI sustained during childhood. Our correlational findings indicating that severity of TBI was associated with reduction in hippocampal head volume suggests that severity of injury might mediate the relation between hippocampal head volume and memory performance. One limitation of this investigation is that our sample of TBI patients is modest and prohibits a full mediation analysis. However, investigation of the effects of severity of pediatric TBI on the relation between development of the hippocampal formation and memory during the childhood years remains a promising avenue of research.

An additional limitation of this investigation is that the measure of memory was restricted to subtests of the TOMAL2. To fully understand the relation between memory and hippocampal volume following TBI, it is important to include measures of episodic memory that require retrieval of rich contextual detail, as well as measures evaluating autobiographical memory. Previous research indicates that many individuals show difficulty encoding and retrieving autobiographical events following TBI (e.g., Knight & O'Hagan, 2009). However, it is unclear how autobiographical memory progresses in the years following a TBI sustained in childhood, or if there is a relation between autobiographical memory and hippocampal structure following TBI.

A final limitation of this research is that our analyses reflect brain and behavioral data collected at a single time point 6‐weeks following injury. Considering the results of Thaler et al. (2011) showing behavioral differences on all TOMAL2 measures at 7–10 months postinjury, it is possible that group differences between hippocampal‐memory relations would be increasingly evident as time since the injury progressed. An investigation with a longitudinal design is required to fully elucidate the relationship between hippocampal structural maturation and memory development following TBI.

## 5 CONCLUSION

This study highlights the relation between hippocampal structure and memory function during the subacute stage of mild to severe TBI recovery. Results indicate that segmentation of the hippocampus into head, body, and tail regions might be a more sensitive method for detecting injury‐related volumetric differences in patients with a history of TBI. These results also provide evidence that TBI sustained during childhood results in changes in the relation between brain development and behavioral outcomes. Long‐term deficits in verbal memory are a common manifestation of TBI and these results indicate that injury to the hippocampal formation as a contributing factor to memory dysfunction. Furthermore, these results suggest that disruption to normal brain development and specifically memory‐related development is likely one source of TBI's negative impact on academic success.

## CONFLICT OF INTEREST

None declared.
