# Supplementary material for: Memory and the hippocampal formation following pediatric traumatic brain injury
Source: Brain Behav. 2017 Nov 2;7(12):e00832. doi: 10.1002/brb3.832 (PMC5745237; doi:10.1002/brb3.832)
Supplement: Supplementary file 1 [file BRB3-7-e00832-s001.docx]

Supplementary Table 1. Mean and standard error of A) Left and B) Right hippocampal head, body, and tail volume.

| Subregion | Group | | | | | | Statistic  (*p-*Value) | | |
| --- | --- | --- | --- | --- | --- | --- | --- | --- | --- |
|  | **Traumatic Brain Injury** | | **Extracranial**  **Injury** | | **Typically Developing** | |  |  |  |
|  | n=62 | | n=29 | | n=38 | | TBI vs. EI | TBI vs. TDC | EI vs. TDC |
| Left | *M* | *SE* | *M* | *SE* | *M* | *SE* |  |  |  |
| Head | 1666 | 38 | 1711 | 57 | 1554 | 50 | 1.000 | 0.234 | 0.132 |
| Body | 1379 | 32 | 1377 | 47 | 1546 | 41 | 1.000 | **0.005*** | **0.028*** |
| Tail | 631 | 21 | 620 | 31 | 660 | 28 | 1.000 | 1.000 | 1.000 |
| Total | 3675 | 48 | 3708 | 72 | 3760 | 63 |  |  |  |
|  | **Traumatic Brain Injury**  n=62 | | **Extracranial**  **Injury**  n=29 | | **Typically Developing**  n=38 | | TBI vs. EI | TBI vs. TDC | EI vs. TDC |
| Right | *M* | *SE* | *M* | *SE* | *M* | *SE* |  |  |  |
| Head | 1791 | 37 | 1822 | 55 | 1743 | 48 | 1.000 | 1.000 | 0.856 |
| Body | 1321 | 34 | 1211 | 50 | 1432 | 44 | 0.219 | 0.143 | **0.005*** |
| Tail | 613 | 17 | 640 | 26 | 629 | 23 | 1.000 | 1.000 | 1.000 |
| Total | 3724 | 48 | 3673 | 71 | 3804 | 62 |  |  |  |

Notes: Values adjusted for maternal education, age, total brain volume, and scanner change with difference significant at the .05 level. Bonferroni adjustment for multiple comparisons.
