# Supplementary material for: Memory and the hippocampal formation following pediatric traumatic brain injury
Source: Brain Behav. 2017 Nov 2;7(12):e00832. doi: 10.1002/brb3.832 (PMC5745237; doi:10.1002/brb3.832)
Supplement: Supplementary file 2 [file BRB3-7-e00832-s002.docx]

| **Group** | **Left Hippocampus** | | | **Right Hippocampus** | | |
| --- | --- | --- | --- | --- | --- | --- |
|  | Head | Body | Tail | Head | Body | Tail |
| **Visual Selective Reminding** | | | |  |  |  |
|  |  |  |  |  |  |  |
| **Traumatic Brain Injury** | .09 | -.11 | -.18 | .16 | -.10 | -.13 |
| **Extracranial Injury** | -.06 | -.22 | .21 **‡** | -.30 **‡** | -.08 | .18 |
| **Typically Developing** | **.**09 | -.21 | .12 | -.03 | -.12 | .04 |
| **Word Selective Reminding** | | | |  |  |  |
|  |  |  |  |  |  |  |
| **Traumatic Brain Injury** | .28^*^ | -.38^**^ ҂**‡** | -.13 | .24 **‡** | -.40^**^ ҂**‡** | -.03 |
| **Extracranial Injury** | -.05 | .14 | .03 | -.16 | .09 | .15 |
| **Typically Developing** | .21 | -.04 | -.19 | .17 | .08 | -.05 |
| **Delayed Word Selective Reminding** | | | |  |  |  |
|  |  |  |  |  |  |  |
| **Traumatic Brain Injury** | .23 | -.26^*^ | -.05 | .25^*^ | -.22 | -.06 |
| **Extracranial Injury** | .31 | -.45^*^ | .36 **‡^†^** | .01 | -.14 | .43^*^ **‡^†^** |
| **Typically Developing** | .21 | -.15 | -.33^*^ ҂ | .36^*^ | -.19 | -.12 |

Supplemental Table 2. Pearson correlation for performance on Test of Memory and Learning – Second Edition (TOMAL2) subtests and Left and Right hippocampal head, body, and tail volume.

Note: Values adjusted for maternal education, age, total brain volume, and scanner change.

^*^*p* < .05; ^**^*p* < .01. Fisher’s r to z transform, p < .05. ҂ TBI ≠ TDC, **‡** TBI ≠ EI, **^†^** EI ≠ TDC.
